# Programming Effects of Prenatal Glucocorticoid Exposure with a Postnatal High-Fat Diet in Diabetes Mellitus

**DOI:** 10.3390/ijms17040533

**Published:** 2016-04-08

**Authors:** Jiunn-Ming Sheen, Chih-Sung Hsieh, You-Lin Tain, Shih-Wen Li, Hong-Ren Yu, Chih-Cheng Chen, Miao-Meng Tiao, Yu-Chieh Chen, Li-Tung Huang

**Affiliations:** 1 Department of Pediatrics, Chang Gung Memorial Hospital-Kaohsiung Medical Center, Chang Gung University, College of Medicine, Kaohsiung 833, Taiwan; ray.sheen@gmail.com (J.-M.S.); tainyl@hotmail.com (Y.-L.T.); violet7053@gmail.com (S.-W.L.); yuu2004taiwan@gmail.com (H.-R.Y.); charllysc@yahoo.com.tw (C.-C.C.); tmm@adm.cgmh.org.tw (M.-M.T.); 2 Department of Medical Administration, Pu-Li Christian Hospital, Pu-Li, Nantou 545, Taiwan; hcch.d201@gmail.com; 3 Department of Applied Chemistry, National Chi-Nan University, Pu-Li, Nantou 545, Taiwan

**Keywords:** diabetes mellitus, high fat diet, prenatal dexamethasone exposure, programming

## Abstract

Increasing evidence has shown that many chronic diseases originate from early life, even before birth, through what are termed as fetal programming effects. Glucocorticoids are frequently used prenatally to accelerate the maturation of the lungs of premature infants. High-fat diets are associated with insulin resistance, but the effects of prenatal glucocorticoid exposure plus a postnatal high-fat diet in diabetes mellitus remain unclear. We administered pregnant Sprague-Dawley rats’ intraperitoneal dexamethasone (0.1 mg/kg body weight) or vehicle at gestational days 14–20. Male offspring were administered a normal or high-fat diet starting from weaning. We assessed the effects of prenatal steroid exposure plus postnatal high-fat diet on the liver, pancreas, muscle and fat at postnatal day 120. At 15 and 30 min, sugar levels were higher in the dexamethasone plus high-fat diet (DHF) group than the vehicle plus high-fat diet (VHF) group in the intraperitoneal glucose tolerance test (IPGTT). Serum insulin levels at 15, 30 and 60 min were significantly higher in the VHF group than in the vehicle and normal diet group. Liver insulin receptor and adenosine monophosphate-activated protein kinase mRNA expressions and protein levels were lower in the DHF group. Insulin receptor and insulin receptor substrate-1 mRNA expressions were lower in the epididymal adipose tissue in the VHF and DHF groups. “Programming” of liver or epididymal adipose tissue resulted from prenatal events. Prenatal steroid exposure worsened insulin resistance in animals fed a high-fat diet.

## 1. Introduction

There is more and more evidence showing that many chronic diseases originate from early life, even before birth, through what are called “programming” effects [1]. Barker *et al.* [2,3] found that low birth weight infants had higher probability of developing metabolic disorders later in life, including insulin resistance and glucose intolerance.

Glucocorticoids are frequently used prenatally to accelerate the maturation of the lungs in the premature babies who were highly fragile because of their lower antioxidant defense in this oxidative environment and glucocorticoids can decrease the damage from the oxidative environment [4]; however, there is controversy regarding the long-term effects of this treatment [5]. Dalziel *et al.* [6] reported that offspring with prenatal exposure to betamethasone might develop insulin resistance at 30 years of age. The underlying mechanisms remain unclear.

Feeding behavior can also be programmed by prenatal stress caused by decreased placenta expression of 11β-hydroxysteroid dehydrogenase type-2 [7], which plays a key role in regulating glucocorticoid hormones [8]. Furthermore, altered expression of adipocyte proteins in response to maternal undernutrition has been reported, e.g., adipocytes of antenatal nutrient-restricted lambs had increased expression of 11β-hydroxysteroid dehydrogenase type-1. This may lead to increased cortisol exposure and adipocytes proliferation [9]. In addition, early nutrient restriction in sheep was reported to increase the expression of both 11β-hydroxysteroid dehydrogenase type-1 and glucocorticoid receptor [10]. Moreover, rat offspring from dams who suffered from variable stress, e.g., restraint, swim, cold exposure, group housing and light on during dark phase during the third week of gestation were more susceptible to obesity when weaned on a high fat diet; this susceptibility may have been related to excessive exposure of the developing fetus to maternal glucocorticoid [11]. Type 2 diabetes mellitus has two major features: desensitization of peripheral target tissues/organs to the actions of insulin, *i.e.*, insulin resistance, and insufficient response of β-cell to glucose stimuli. It is well known that high-fat diets can cause insulin resistance [12,13]. A combination of these two factors is likely to the cause diabetes mellitus in rat offspring.

In this study, we evaluated whether rats treated prenatally with dexamethasone plus a high-fat diet show deficits in glycemic homeostasis. In addition, we examined the effects of these treatments on the expression of genes important in glucose and fatty acid metabolism in the liver, pancreas, muscle and adipose depots.

## 2. Results

**Figure 1:**
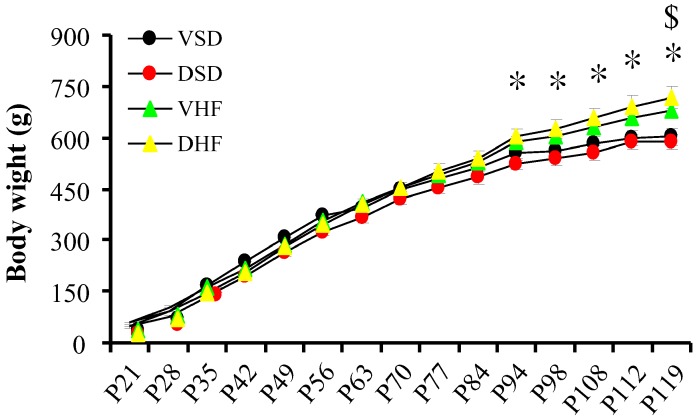
Body weights. Mean body weight from weaning until six months. Groups were compared by repeated measures Analysis of Variance (ANOVA) with *post hoc* least significant difference testing. *, vehicle with normal diet (VSD) group *vs.* vehicle and high-fat diet (VHF) group, *p* < 0.05; $, dexamethasone and normal diet (DSD) group *vs.* dexamethasone and high-fat diet (DHF) group, *p* < 0.05.

The birth body weight was lighter in the prenatal steroid exposure group than in the vehicle control group (5.9 ± 0.1 *vs.* 8.0 ± 0.2 g, *p* = 0.001). There was no difference in body weight from weaning among the four groups until from 94 days of postnatal age (P94), when the body weight was heavier in the vehicle and high-fat diet (VHF) group than in the vehicle with normal diet (VSD) group. The dexamethasone and high-fat diet (DHF) group had a higher body weight than the dexamethasone and normal diet (DSD) group at P119. The high-fat diet had a positive effect on body weight increase, while prenatal steroid did not significantly influence the body weight. The mortality rates of the four groups of animals were all 0% (Figure 1).

### Intraperitoneal Glucose Tolerance Test (IPGTT) and Insulin Tolerance Test (ITT)

**Figure 2:**
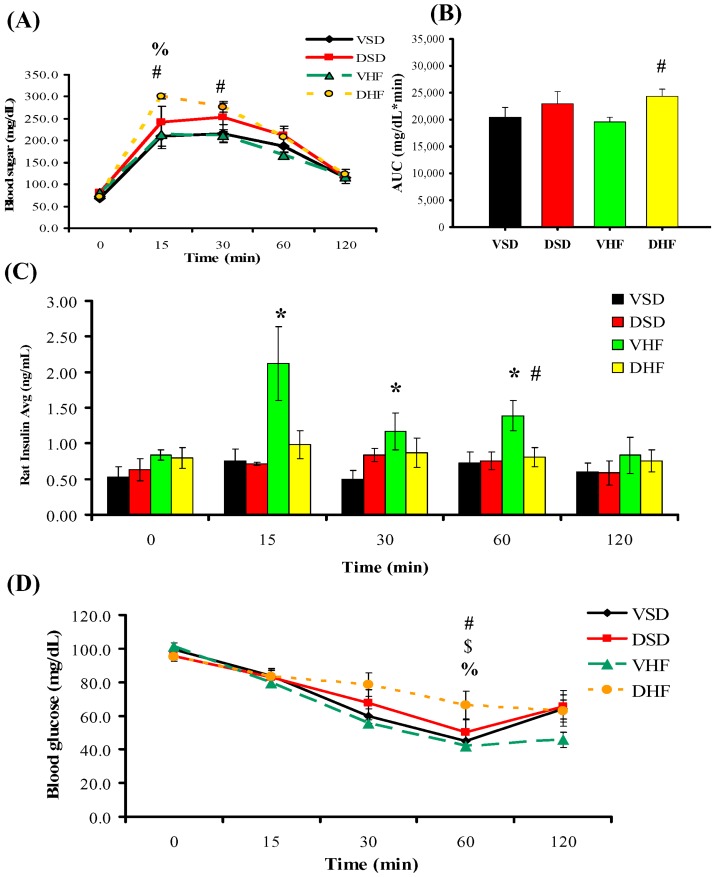
Glucose and insulin levels post-stimulation tests: (**A**) intraperitoneal glucose tolerance test; (**B**) glucose area under curves (AUC); (**C**) insulin levels; and (**D**) insulin tolerance test. Data were analyzed by repeated measures ANOVA with *post hoc* least significant difference testing. *, VSD *vs.* VHF, *p* < 0.05; #, VHF *vs.* DHF, *p* < 0.05; %, VSD *vs.* DHF, *p* < 0.05; $, DSD *vs.* DHF, *p* < 0.05.

Sugar levels at 15 and 30 min were higher in the DHF group than in the VHF group and at 15 min in the DHF group than in the VSD group (Figure 2A). The glucose area under curve (AUC) was larger in the DHF group than in the VHF group (Figure 2B). Serum insulin levels at 15, 30 and 60 min after intraperitoneal glucose injection were significantly higher in the VHF group than in the VSD group (Figure 2C). The glucose level at 60 min during the ITT was higher in the DHF group than the other three groups (Figure 2D).

To investigate the mechanisms underlying the development of insulin resistance after prenatal glucocorticoid overexposure plus postnatal high fat-diet, we measured the transcript levels of genes involved in glucose metabolism in the liver, muscle and fat depots.

**Figure 3:**
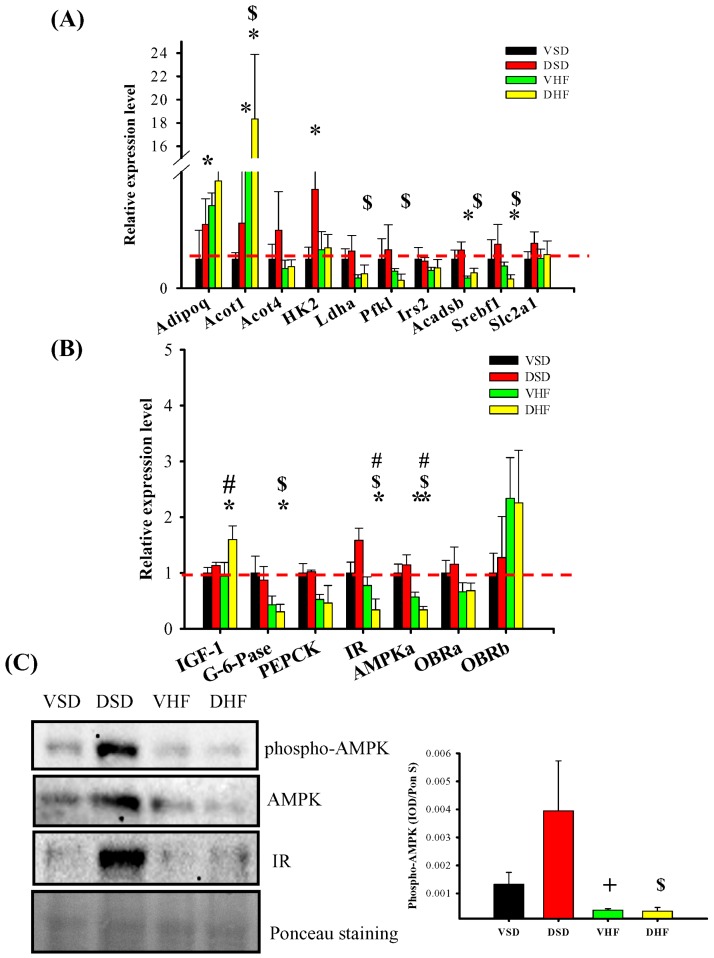
Hepatic mRNA transcript levels and adenosine monophosphate-activated protein kinase (AMPK) and phsopho-AMPK protein levels: (**A**,**B**) hepatic mRNA transcript levels; and (**C**) hepatic AMPK and pAMPK protein levels. The red dotted line represented that the relative gene expression was 1 when compared to VSD group. Adipoq, adiponectin; Acot1, Acyl-CoA thioesterase 1; Acot4, Acyl-CoA thioesterase 4; HK2, Hexokinase 2. Ldha, Lactate dehydrogenase A; Pfk1, 6-phosphofructokinase; Irs2, Insulin receptor substrate 2; Acadsb, Acyl-CoA dehydrogenase; Srebf1, Sterol regulatory element-binding transcription factor 1; Slc2a1, Solute carrier family 2, member 1; IGF1, Insulin-like growth factor 1; G-6-Pase, Glucose 6-phosphatase; PEPCK, phosphoenolpyruvate carboxykinase; IR, Insulin receptor; AMPKa, AMP-activated protein kinase alpha; OBRa, leptin/obese receptor a; OBRb, leptin/obese receptor b. Data were compared by two-way ANOVA followed with *post hoc* least significant difference tests. * *vs.* VSD, *p* < 0.05; $, DSD *vs.* DHF, *p* < 0.05; #, VHF *vs.* DHF, *p* < 0.05; +, DSD *vs.* VHF, *p* < 0.05.

In the liver, the mRNA levels of Acot1, Acadsb and Srebf1 were increased, while the mRNA level for G-6-Pase was decreased in the DHF group compared to that in the DSD group. The mRNA levels of Adiponectin and hexokinase 2 were increased in the DSD group compared to that in the VSD group and the mRNA levels for IGF-1 were increased in the DHF group compared to that in the VHF group. Additionally, the mRNA levels of both insulin receptor (IR) and AMP-activated protein kinase (AMPK) were both decreased in the DHF group compared to that in the other groups. Since AMPK plays a key role in stimulating fatty acid oxidation and suppressing hepatic lipogenesis, decreased AMPK levels may be a mechanism for the increased insulin resistance. The results for AMPK or pAMPK protein levels were consistent with the results of Western blot analysis (Figure 3).

**Figure 4:**
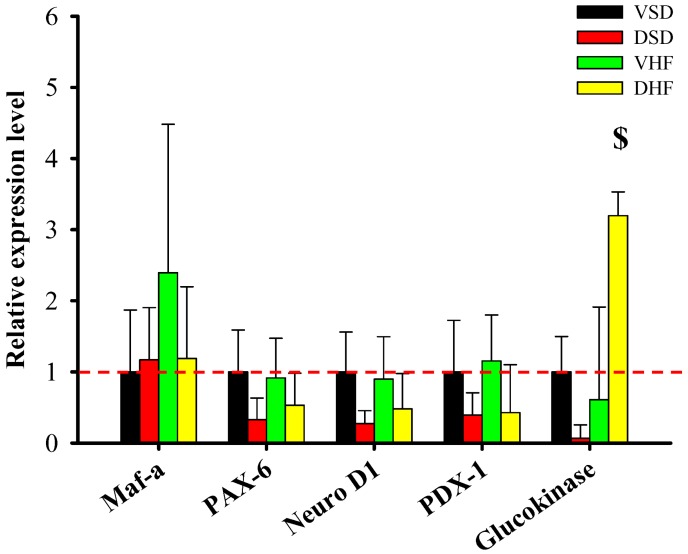
Pancreas mRNA transcript levels. The red dotted line represented that the relative gene expression was 1 when compared to VSD group. Maf-a, V-maf avian musculoaponeurotic fibrosarcoma oncogene homolog A; PAX-6, paired box gene 6; Neuro D1, neuronal differentiation 1; PDX-1, pancreatic and duodenal homeobox factor-1. Data were compared by two-way ANOVA with *post hoc* least significant difference followed by LSD tests. $, DSD *vs.* DHF, *p* < 0.05.

In the pancreas (Figure 4), we found increased glucokinase mRNA expression in the DHF group compared to that in the VSD group. There was no difference in the pdx-1, maf-a, pax-6 and neuro D1 mRNA expression among the four groups.

**Figure 5:**
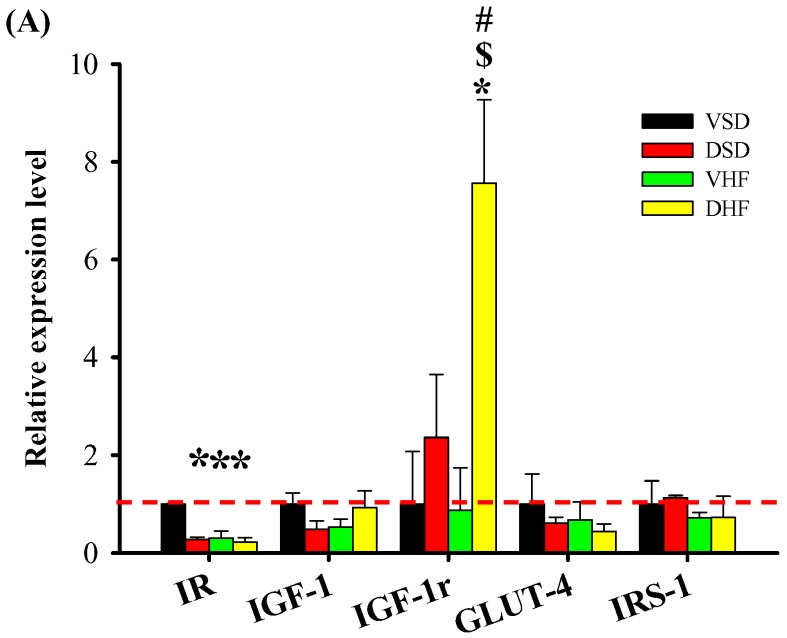

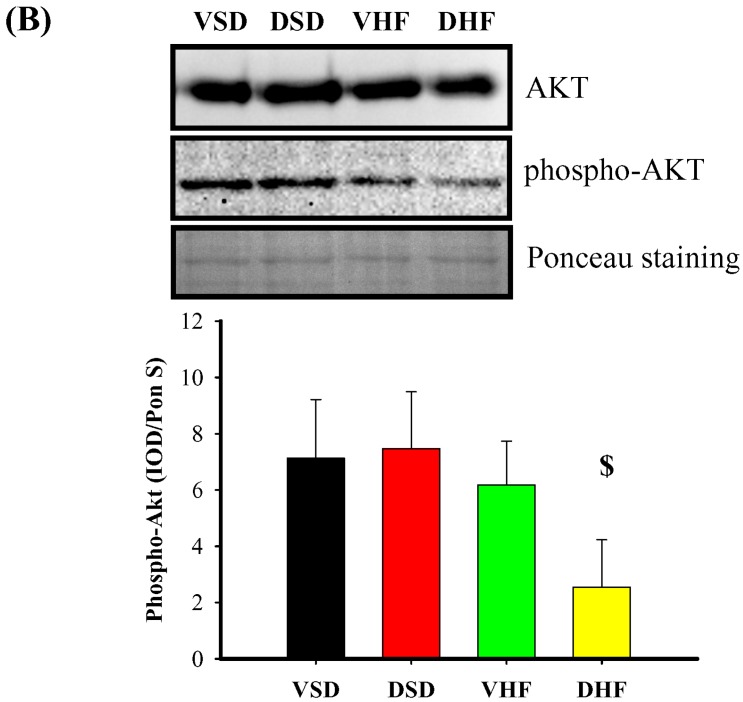
Gastrocnemius muscle mRNA transcript and phopho-AKT protein levels: (**A**) gastrocnemius mRNA transcript levels; and (**B**) gastrocnemius phosphor-AKT protein levels. The red dotted line represented that the relative gene expression was 1 when compared to VSD group. IR, insulin receptor; IGF-1r, insulin-like growth factor 1 receptor; GLUT-4, glucose transporter type 4; IRS-1, insulin receptor substrate 1. Data were compared by two-way ANOVA with *post hoc* least significant difference tests. * *vs.* VSD, *p* < 0.05; $, DSD *vs.* DHF , *p* < 0.05; #, VHF *vs.* DHF, *p* < 0.05.

In gastrocnemius muscle (Figure 5), we observed decreased IR mRNA expression in the DSD, VHF and DHF groups compared to that in the VSD group. IGF1-r mRNA expression was increased in the DHF group compared to that in the other three groups.

**Figure 6:**
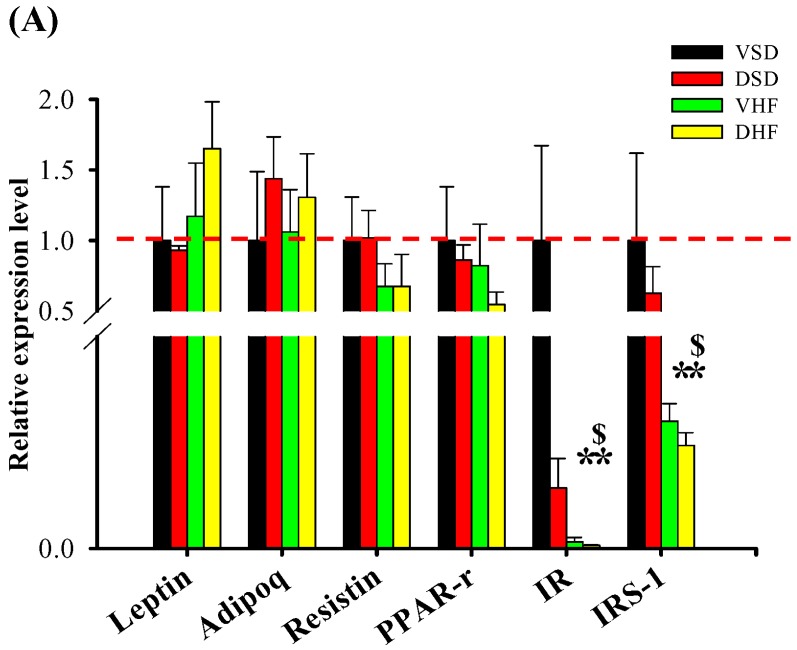

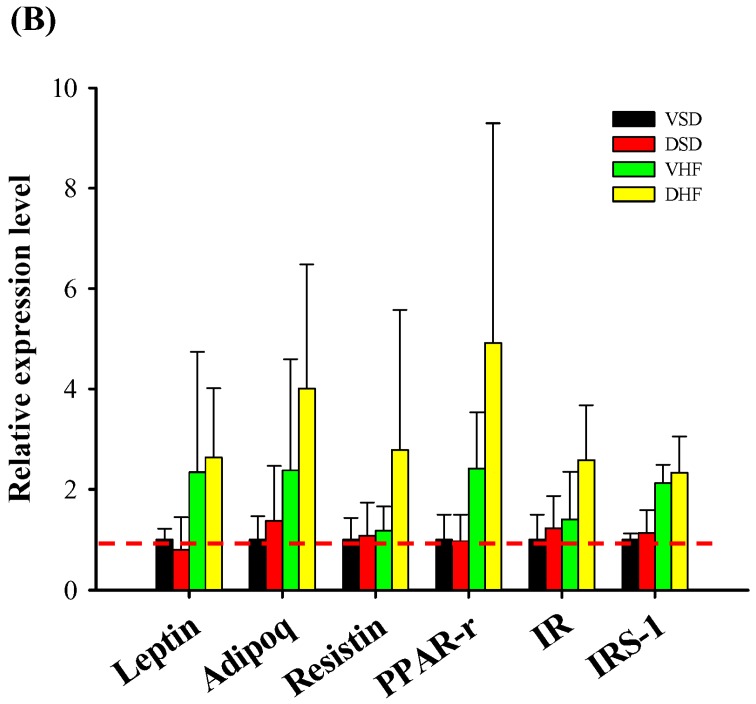
Adipose mRNA transcript levels: (**A**) epididymal fat; and (**B**) omentum fat. The red dotted line represented that the relative gene expression was 1 when compared to VSD group. PPAR-r, Peroxisome proliferator-activated receptor gamma. Data were compared by two-way ANOVA with *post hoc* least significant difference followed by LSD tests. * *vs.* VSD, *p* < 0.05; $, DSD *vs.* DHF , *p* < 0.05.

In the epididymal adipose tissue (Figure 6A), IR and insulin receptor substrate-1 (IRS-1) mRNA expressions were decreased in the VHF and DHF groups. However, there was no difference in omentum fat (Figure 6B). There were no differences in the leptin, adiponectin, resistin and PPAR-r expressions among the four groups in the epididymal adipose tissue or omentum fat.

## 3. Discussion

In this study, we found: (1) higher sugar level at 15 and 30 min in the DHF group than in the VHF group; (2) the serum insulin level at 15, 30 and 60 min were significantly higher in the DHF group than in the VSD group; (3) lower liver IR and AMPK mRNA and protein in the DHF group than the other three groups; and (4) lower IR and IRS-1 mRNA expression in the epididymal adipose tissue in the VHF and DHF groups than the other two groups.

Glucocorticoids can promote gluconeogenesis in the liver and decrease glucose uptake and utilization in the skeletal muscle and white adipose tissue. Excess glucocorticoid exposure causes hyperglycemia by inducing gluconeogenic enzyme genes in the liver. In addition, they also take a role in catecholamine induced glycogenolysis and/or inhibit insulin stimulated glycogen synthesis in the skeletal muscle. Furthermore, they adjust the function of pancreatic α and β cells to regulate the glucagon and insulin secretion [14,15]. Maternal glucocorticoid administration can influence glucose homeostasis in various organs in offspring. Franko *et al.* [16] reported that after maternal injection with dexamethasone, offspring showed higher phosphoenolpyruvate carboxykinase activity in the liver than in controls both at birth and weanlings. We previously found that seven-day-old rats with antenatal glucocorticoid exposure had lower pdx-1, maf-a, neurod-1, and pax-6 mRNA expressions in the pancreas [17]. Blasio *et al.* [18] also demonstrated that maternal glucocorticoid exposure during early pregnancy altered glucose homeostasis and induced hyperinsulinemia in adult male sheep offspring.

Decreases in insulin secretion and insulin sensitivity occur during the development of type 2 diabetes. Our previous study showed prenatal dexamethasone may have programming effects on pancreas development by decreasing PD 120 pancreatic β cell mass with lower serum insulin level at 15 min in IPGTT without differences in sugar levels [18]. Autopsy studies from various populations revealed the pancreatic β cell mass reduced significantly in patients with type 2 diabetes compared to that in nondiabetic individuals [19,20]. A high-fat diet was reported to induce insulin resistance. Therefore, we evaluated the combined effects of prenatal glucocorticoid and a high-fat diet.

First, we evaluated the effects of prenatal glucocorticoid exposure. We found increased adiponectin mRNA levels in the liver of DSD group than in the VSD group. Adiponectin is primarily an adipocyte-derived protein that has anti-obesity, antidiabetic and anti-inflammatory characteristics. Higher circulating adiponectin levels were reported to be associated with a lower risk of type 2 diabetes [21]. The expression of adiponectin in the liver is downregulated in morbidly obese patients with non-alcoholic steatohepatitis compared to that in patients with simple steatosis [22]. We found no difference in sugar levels in the IPGTT between the DSD group and the VSD group. There were also no difference in leptin, adiponectin, and resistin expression among these four groups in the epididymal adipose tissue and omentum fat. Liver adiponectin seemed to play a minor role in this study.

Next, we observed increased IGF-1 mRNA levels in the liver of the DHF group than in the VHF group and increased IGF1-r mRNA expression in the gastrocnemius muscle in the DHF group compared to that in the other three groups. IGF-1 gene expression was reported to be downregulated in liver tissues and progressively decreased with the severity and duration of diabetic state [23]. Increased IGF-1 mRNA levels in the liver may compensate to overcome the hyperglycemic state during the early stage of diabetes. Sugar levels in the DHF group were higher than in the VHF group. The serum insulin level at 60 min after intraperitoneal (i.p.) glucose injection was significantly higher in the DHF group than in the VHF group. The glucose level was higher at 60 min during the ITT in the DHF group than in the other three groups. These data indicate that prenatal glucocorticoid exposure strengthens high-fat induced insulin resistance. Since GLUT4 is a downstream target of the IR and IGF-1r, disruption of the IR signaling pathway in muscle can be compensated functionally by increasing IGF-Ir expression [24].

Additionally, we found IR and AMPK mRNA levels were both decreased in the livers of the DHF group compared to that in the other groups. In gastrocnemius muscle, IR mRNA expression was decreased in the DSD, VHF, and DHF groups compared to that in the VSD group. Phopho-Akt protein levels were decreased in the DHF group compared to that in the DSD group. In the epididymal adipose tissue, we observed decreased IR and IRS-1 mRNA expression in the VHF and DHF groups. However, there were no differences in the omentum fat. The AMPK system takes a major role in regulating glucose metabolism. The mechanism can be through its effects on energy metabolism pathways acutely and gene expression change chronically. The relationship between AMPK activation and advantageous metabolic effects in diabetic rodent models provides a foundation for developing new therapeutic strategies and nutritional use of AMPK activators to prevent or reverse hepatic disorders related to type 2 diabetes and obesity. In this study, we found lower liver AMPK mRNA and protein in the DHF group, indicating that prenatal steroid and postnatal high-fat diet may affect glucose homeostasis through the AMPK pathway [25,26]. In addition, insulin signaling is required for insulin to act both, directly and indirectly, on hepatic glucose production. Lower liver IR was also observed in the DHF group.

Insulin resistance in obesity and type 2 diabetes is characterized by fewer insulin stimulated glucose transport and less metabolism in skeletal muscle and adipocytes. These functional defects may result partly from impaired insulin signaling. In both the muscle and adipocytes, binding of insulin to its receptor, insulin receptor phosphorylation and activation of tyrosine kinase, and IRSs phosphorylation are reduced. There are also tissue-specific alterations. In adipocytes isolated from obese humans with type 2 diabetes, expression of IRS-1 is reduced, followed by IRS-1–associated PI3K activity decreased, then IRS-2 becomes the main docking protein for PI3K [27]. In our study, we detected lower IR and IRS-1 mRNA expression in the epididymal adipose tissue but not in the omentum fat in the VHF and DHF groups. Thus, the roles of IR and IRS-1 in various adipose tissues require further analysis.

## 4. Material and Methods

### 4.1. Animals

This study was approved by the Institutional Animal Care and Use Committee of the Kaohsiung Chang Gung Memorial Hospital (number 2014032607, approved date 2014/05/05). The experiments were done under the Guidelines for Animal Experiments of Chang Gung Memorial Hospital and Chang Gung University (Kaohsiung, Taiwan). We purchased virgin Sprague-Dawley (SD) rats (12–16 weeks old) from the BioLASCO Taiwan Co., Ltd. (Taipei, Taiwan). SD rats were fed in the animal care center with a 12-h light/dark cycle and lights on at 7 a.m. We allowed SD female rats to mate with male rats for 24 h. After one day, the female rats were separated from the male rats individually. Pregnant females were randomly divided into prenatal steroid exposure and control groups.

### 4.2. Prenatal Steroid Exposure and Postnatal High Fat Diet

We administered pregnant SD rats in the prenatal steroid exposure group i.p. dexamethasone (0.1 mg/kg/day) from gestational days (GD) 14–20. The vehicle control group received normal saline i.p. at GD 14–20. We designated the day of birth as postnatal Day 0 (PD 0). Pups were weaned at PD 21, and had free access to standard chow and water. Only the male offspring were used for this study.

Four groups of rats were used: the number in each group was ten.

1. VSD group: Offspring of SD rats that received i.p. normal saline at GD 14–20; received normal diet after weaning, sacrificed at 4 months of age.
2. DSD group: Offspring of SD rats that received i.p. dexamethasone at GD 14–20; received normal diet after weaning, sacrificed at 4 months of age.
3. VHF group: Offspring of SD rats that received i.p. normal saline at GD 14–20; received high-fat diet (58% high-fat diet, Research Diet, D12330, soybean Oil 25 gm%, coconut Oil, hydrogenated 333.5 gm%) (Supplementary Table S1) from weaning, sacrificed at 4 months of age.
4. DHF group: Offspring of SD rats that received i.p. dexamethasone at GD 14–20; received high-fat diet from weaning, sacrificed at 4 months of age.

### 4.3. I.P. Glucose Tolerance Test (IPGTT) and Insulin Tolerance Test (ITT)

After an 8-h fast at PD 110, blood samples were collected at five time points: before injection and at 15, 30, 60, and 120 min after the i.p. injection of glucose (2 g/kg body weight). Plasma glucose levels were immediately measured using the enzymatic (hexokinase) method with a glucose assay kit. Serum insulin levels were checked using enzyme -linked immunosorbent assay (Crystal Chem Inc., Downers Grove, IL, USA) The ITT was performed by injecting insulin i.p. (1 U/kg body weight) after a 5-h fast. We measured blood glucose levels before and 15, 30, 60, and 120 min after insulin injection using methods as described above.

### 4.4. Quantitative Real-Time Polymerase Chain Reaction (PCR) Analysis

We extracted RNA by using TRI Reagent then treated with DNase I to measure mRNA expression. RNA (2 µg) was reverse-transcribed with random primers in a total volume of 40 µL as previously published [28]. We performed control RT reactions by omitting the RT enzyme followed by PCR amplification. We conducted two-step quantitative real-time PCR using Quantitect SYBR Green PCR Reagents according to the manufacturer’s protocol on a LightCycler Real-Time PCR System (Roche Diagnostics Ltd., Basel, Switzerland). The β-Actin served as a control housekeeping gene. The primers used are listed in Table 1. All samples were run in duplicate (2.5 µL of cDNA/well in a 96-well format). The comparative threshold cycle (*C*_t_) method was employed for the relative quantification of gene expression. The averaged *C*_t_ was subtracted from the corresponding averaged β-Actin value for each sample to result in ∆*C*_t_. We determined ∆∆*C*_t_ by subtracting the average control ∆*C*_t_ value from the average experimental ∆*C*_t_. The fold increase was established by calculating 2^−∆∆*C*t^ for experimental *versus* control samples.

### 4.5. Western Blot

Western blot analysis was performed as previously described [28]. The following antibodies were used: AMPK (goat anti-rat antibody (1:1000, overnight incubation; Santa Cruz Biotechnology, Santa Cruz, CA, USA); phospho-AMPK antibody (rabbit anti-rat antibody (1:1000, overnight incubation; Santa Cruz Biotechnology) and phospho-Akt (Ser 473) antibody (rabbit anti-rat antibody (1:1000, overnight incubation; Cell Signaling Technology, Danvers, MA, USA)). We used Super Signal West Pico reagent (Pierce; Rockford, IL, USA) to visualize bands of interest and quantified them using densitometry as integrated optical density, factored for Ponceau S red (PonS) staining to correct variations in total protein loading. Protein levels were represented as integrate optical density/PonS.

### 4.6. Statistical Analyses

The results are presented as the mean ± standard error of the mean. We analyzed data by two-way ANOVA with *post hoc* least significant difference test. All analyses were carried out using the Statistical Package for the Social Sciences (SPSS) software version 15 (SPSS Inc., Chicago, IL, USA) on a PC-compatible computer. Significance was defined as *p* < 0.05 for all tests.

## 5. Conclusions

In conclusion, we found evidence for “programming” of the liver or of epididymal adipose tissue mass by prenatal events in animal model. Exposure to a high-fat diet was associated with worsening of insulin resistance in animals exposed to excess glucocorticoid *in utero*.

## Figures and Tables

**Table 1:** Primers used in real-time polymerase chain reaction.

Gene	Sense (5′–3′)	Anti-Sense (3′–5′)
Adipocytokine signaling pathway		
*Adiponectin*	GGAGACGCAGGTGTTCTTGG	AGCCCTACGCTGAATGCTGA
*Leptin*	CGGTTCCTGTGGCTTTGGT	CCGACTGCGTGTGTGAAATG
*OBRa*	CCTCTTGTGTCCTGCTGCTCGG	TTCTATGGACTGTTGGGAGGTTGGT
*OBRb*	GCATGCAGAATCAGTGATATTTGG	CAAGCTGTATCGACACTGATTTCTTC
*Slc2a1*	GCTCCATTTAGGATTCGCCCA	TATACACAGCAGGGCAGGAGT
*Resistin*	TCATGCCCAGAACCGAGTTG	CAGCCCCAGGACAAGGAAGA
*Visfatin*	TCTGGAAATCCGCTCGACAC	CACTCCGTCCCCTTGAATGA
Fatty acid metabolism		
*Acadsb*	GGACTGGCCCAAGGATGTTT	ATAAATGGCCTCCCGGCTTC
*Acot1*	GTGATGGTTTTGGCAGGAAAAGT	AATGTGCTCTTTTCCCTTACAGC
*Acot4*	TTGCCATCTCAATGGGGTAGAT	AGGGAGTCTCTCTTAACGTTTACC
*Perilipin1*	GAGGGGCTGATCTGGCTTTG	GCATCTTTTGCCGTCCTGAA
*PPAR-r*	GGCTTCATGACAAGGGAGTTTC	AACTCAAACTTGGGCTCCATAAAG
Glycolysis/Gluconeogenesis		
*G6Pase*	AACGTCTGTCTGTCCCGGATCTAC	ACCTCTGGAGGCTGGCATTG
*Ldha*	TCAGCGTCCCATGTATCCTG	CTGGACCAACTGGACTAACCA
*PEPCK*	CTCACCTCTGGCCAAGATTGGTA	GTTGCAGGCCCAGTTGTTGA
*Pfkl*	CTTACCGATCACCCTCGTTC	CCACAGGTGCTCTGTTCTGA
Insulin/IGF-1 signaling pathway		
*AMPKa*	GTCGGCACCTTCGGCAAAGTGAA	AGAAATTCACCATCTGACATCATATTAGA
*Glucokinase*	CAGTGGAGCGTGAAGACAAA	AGGGAAGGAGAAGGTGGAGC
*GLUT-4*	TTTCCAGTATGTTGCGGATG	TCAGTCATTCTCATCTGGCC
*Hexokinase 2*	ATGGTCCTCCCCCACTCTAC	TCCCACCCAACATCTACCTC
*IR*	TCGAACCCTTCCTAACAG	CAGGTCCAAAGACAAACAGA
*IRS-1*	ATCTTCCTTTGGCGCAGCTA	CAGCACGAAAAAGCGCTTA
*IRS-2*	GAATCCCCCAGGGACAGTAG	GGGGAGGGGGAGTTTAGTGT
*IGF-1*	TCAGTTCGTG TGTGGACCAG	TCACAGCTCCGGAAGCAAC
*IGF-1r*	TGGCAGAACTGCTGTCTGAG	AACGCAGGGTCTAGTTGAGC
*SREBF1*	GCTGATGGAGACAGGGAGTT	GCAGTTGATGTAGAGGCTAAGC
Insulin secretion		
*Maf-a*	GGAGGTCATCCGACT GAAACA	CCGCCAACTTCTCGTATTTCTC
*Neuro D1*	CTCGCTGTGA GATCC CCATAG	TAATCGTGAAAGATGGCATTAAGC
*PAX-6*	CTCCTCGTACTCCTGCATGCT	GGGCTGACTGTTCATGTGTGTT
*Pdx-1*	GCTGGAGCTGGAGAAGGAAT	CGTTGTCCCGCTACGTT
*β-Actin*	TACTGCCCTGGCTCCTA	GGGCCGGACTCATCGTA

## Supplementary Materials

Supplementary materials can be found at http://www.mdpi.com/1422-0067/17/4/533/s1.
