# Supplementary material for: Programming Effects of Prenatal Glucocorticoid Exposure with a Postnatal High-Fat Diet in Diabetes Mellitus
Source: Int J Mol Sci. 2016 Apr 8;17(4):533. doi: 10.3390/ijms17040533 (PMC4848989; doi:10.3390/ijms17040533)
Supplement: Supplementary file 1 [file ijms-17-00533-s001.pdf]

# Supplementary Materials: Programming Effects of Prenatal Glucocorticoid Exposure with a Postnatal High-Fat Diet in Diabetes Mellitus

**Jiunn-Ming Sheen, Chih-Sung Hsieh, You-Lin Tain, Shih-Wen Li, Hong-Ren Yu, Chih-Cheng Chen, Miao-Meng Tiao, Yu-Chieh Chen and Li-Tung Huang**

**Table S1.** Nutritional composition of high fat (HFD) and standard diets.

| HFD (D12330) | Gm % | Kcal % | Standard Diet | Gm % | Kcal % |
|---|---|---|---|---|---|
| Protein | 23.0 | 16.4 | Protein | 16.8 | 16.4 |
| Carbohydrate | 35.5 | 25.5 | Carbohydrate | 74.3 | 73.1 |
| Fat | 35.8 | 58.0 | Fat | 4.8 | 10.5 |
| Total | – | 100 | Total | – | 100 |
| Kcal/gm | 5.56 | – | Kcal/gm | 4.07 | – |
| Ingredients | gm | kcal | Ingredients | gm | kcal |
| Casein, 30 Mesh | 228 | 912 | Casein, 30 Mesh | 228 | 912 |
| DL-Methionine | 2 | 0 | DL-Methionine | 2 | 0 |
| Maltodextrin 10 | 170 | 680 | Maltodextrin 10 | 170 | 680 |
| Corn Starch | 175 | 700 | Corn Starch | 835 | 3340 |
| Sucrose | 0 | 0 | Sucrose | 0 | 0 |
| Soybean Oil | 25 | 225 | Soybean Oil | 25 | 225 |
| Coconut Oil, Hydrogenated | 333.5 | 3001.5 | Coconut Oil, Hydrogenated | 40 | 360 |
| Mineral Mix S10001 | 40 | 0 | Mineral Mix S10001 | 40 | 0 |
| Sodium Bicarbonate | 10.5 | 0 | Sodium Bicarbonate | 10.5 | 0 |
| Potassium Citrate, 1 $H_2O$ | 4 | 0 | Potassium Citrate, 1 $H_2O$ | 4 | 0 |
| Vitamin Mix V10001 | 10 | 40 | Vitamin Mix V10001 | 10 | 40 |
| Choline Bitartrate | 2 | 0 | Choline Bitartrate | 2 | 0 |
| FD&C Blue Dye #1 | 0.05 | 0 | FD&C Yellow Dye #5 | 0.1 | 0 |
| FD&C Red Dye #4 | 0.05 | 0 | | | |
| Total | 1000.1 | 5558.5 | | 1366.6 | 5557 |
